# Male androgenetic alopecia^☆^

**DOI:** 10.1016/j.abd.2024.08.004

**Published:** 2025-01-13

**Authors:** Gabriel Lazzeri Cortez, Karime Hassun, Luciana Ribeiro Patricio Linhares, Verena Florenço, Maria Valeria Bussamara Pinheiro, Mauricio Mendonça do Nascimento

**Affiliations:** a Postgraduate Program in Translational Medicine, Department of Medicine, Universidade Federal de São Paulo, São Paulo, SP, Brazil; b Department of Dermatology, Escola Paulista de Medicina, Universidade Federal de São Paulo, São Paulo, SP, Brazil

**Keywords:** Alopecia, Dustasteride, Finasteride, Minoxidil, Therapeutics

## Abstract

Male androgenetic alopecia (MAA) is quite common and worsens with age, with a significant impact on quality of life, and is increasingly a reason for consultation with a dermatologist. The etiopathogenesis of MAA is multifactorial and genetic and hormonal influences stand out. MAA starts with the process of follicular miniaturization in diverse phenotypic patterns. The diagnosis of MAA is basically clinical and currently corroborated by well-established trichoscopic findings. Despite this, therapeutic options are limited, especially when one considers medications with a high level of scientific evidence. This review aims to help the general dermatologist towards a better understanding of MAA providing a basis for good individualized and judicious therapeutic decisions.

## Introduction and history

Male androgenetic alopecia (MAA) is a common condition that affects a large proportion of men. In ancient Egypt and classical Greece (12^th^ century BCE – 2^nd^ century CE), important and wise individuals were depicted as hairless. Hindu, Catholic, Muslim and Buddhist religious leaders had their hair cut short as a sign of humility and purity.^1^

Throughout history, long hair has become a biological sign of good health, and biblical and mythological heroes and kings have been depicted with thick hair. The 20^th^ century has amplified this concept in the sense that MAA is perceived as negative or undesirable, in part due to the widespread availability of anti-hair loss products and in part due to the explosion of media outlets for cultural dissemination and negative propaganda – radio, cinema, television and the internet.^2^ A search through all children programs in the United States in 2006 showed that only 3% of the characters evaluated were bald. Bald characters in television or cinema tend to be villains or elderly.^3^ In a study of 5,000 images of men in popular magazines published between 2011 and 2012, only 8% were bald.^4^

Hamilton, while studying eunuchs and eunuchoids, found that their brothers developed baldness while the former were protected from alopecia. This observation led to the theory that this endocrine condition could be the cause of MAA.^5^

## Epidemiology

MAA usually manifests itself from the second decade of life on and affects 30% of men around the age of 30, 50% at the age of 50, and tends to worsen with age.^6^, ^7^ In a study conducted in Australia with men aged between 40 and 69 years, it was observed that, for advanced cases of baldness, the prevalence increased with age, going from 31% between 40 and 55 years to 53% in the group aged between 65 and 69 years.^8^ Data from an American study showed that 53% of men between the ages of 40 and 49 had moderate to severe baldness.^9^ Corroborating the information that the incidence increases with age, a study of men from Singapore showed a prevalence of 100% after the age of 80 years.^10^

Different epidemiological data are observed in different ethnic groups, with MAA being four times more frequent in Caucasians when compared to black Americans. Japanese people usually show the onset of the disease up to 10 years later than Caucasians,^11^ while Chinese people usually have a lower incidence in comparison to other ethnicities.^10^

## Etiopathogenesis

The etiopathogenesis of MAA is multifactorial and involves genetic, hormonal and environmental factors. It is important to understand the follicular cycle because MAA is a disease determined by an alteration of this cycle and by follicular miniaturization. More recently, the role of micro-inflammation in its etiology has also been discussed.^12^

### Follicular cycle

Each hair strand is formed in a hair follicle that goes through three phases divided didactically into anagen (growth phase), catagen (involution phase), and telogen (latent or resting phase). At the end of the telogen phase, the hair follicle restarts the anagen phase and a new shaft begins to be produced, even before the previous one is detached. The detachment of the shaft is also known as teloptosis or exogenous phase.

**Fig. 1:**
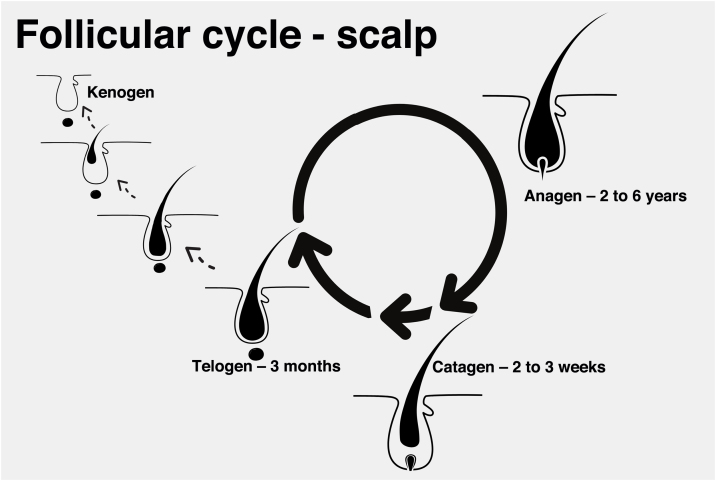
Scalp follicular cycle: anagen phase (cell proliferation), catagen (involution) and telogen (resting). After successive cycles, the kenogen phase (without hair production) may occur.

Exceptionally, the follicle may enter a period of inactivity, in which it is empty for a period of time (kenogen phase). This phase may be present physiologically, but it becomes more common and longer in older patients or those with advanced androgenetic alopecia (Fig. 1).^13^

In MAA, there is a reduction in the anagen phase with premature entry into the catagen phase and a reduction in the anagen-telogen ratio. With a greater predominance of telogen hairs, the patient may notice an increase in hair loss. Some studies suggest that entry into the catagen phase is a consequence of decreased expression of factors that maintain the anagen phase, such as insulin-like growth factor (IGF-1), basic fibroblast growth factor (bFGF) and vascular endothelial growth factor (VEGF) and increased expression of cytokines that promote cell apoptosis, such as transforming growth factor beta 1 (TGF-β1), interleukin-1 alpha (IL-1α) and tumor necrosis factor alpha (TNF-α).^14^

In addition to the change in the follicular cycle, there is also a morphological change in the follicles and, consequently, in the hair shafts: miniaturization. The miniaturized hair is very similar to a vellus hair, with a thickness of less than 0.03 mm.^15^

Miniaturization probably occurs at some point between the catagen phase and the beginning of the next anagen phase, when the dermal papilla is moving and, therefore, more susceptible to external influences.^16^ The hair shaft diameter is determined by the size of the dermal papilla, and the reduction in papillary volume, by mechanisms involving the loss of regulation between pro- and anti-apoptotic agents, may be related to this miniaturization process.^17^

### Genetics

Because it is such a common disease, it is difficult to establish a genetic inheritance pattern for MAA.^12^ Evidence of the importance of the hereditary factor in the development of MAA is supported by the fact that, rarely the disease has already been identified in prepubertal children and these cases of early onset have a strong positive family history for the condition.^18^ Moreover, there is greater concordance of MAA in monozygotic twins, compared to dizygotic ones.^19^

Alterations in the androgen receptor (AR) gene on chromosome Xq12 can lead to increased expression of this receptor and a consequent tendency towards MAA,^20^ but they are not sufficient to cause baldness. The local and systemic presence of androgens also exerts an influence and can be genetically determined, indicating a probable polygenic inheritance in disease development.^21^

This polygenic inheritance model and variable expressivity can explain the different phenotypes and the variation in their age of onset.^22^ There are two important studies in the field of the MAA genome, with currently 10 susceptibility loci identified: 2012;8.1p36.22, 2q37.3, 7p21.1, 7q11.22, 17q21.31, 18q21.123, 2q35, 3q25.1, 5q33.3 and 12p12.1.^23^, ^24^

### Hormonal factor

Estrogen, thyroid hormones, glucocorticoids, retinoids, prolactin and growth hormone interfere with hair growth, but androgens are the most important hormones.^25^

**Fig. 2:**
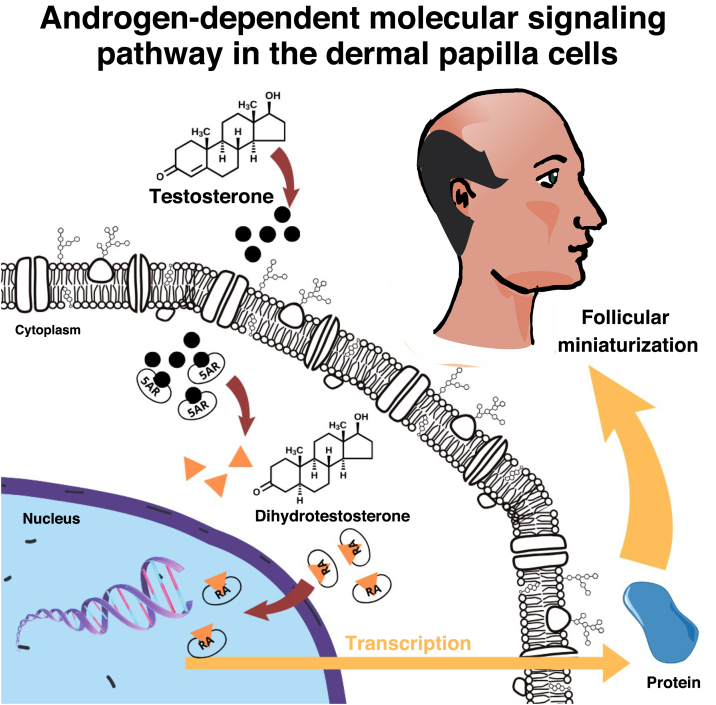
Androgen-dependent signaling pathway in the dermal papilla cells.

The skin and the pilosebaceous unit have the capacity to metabolize and convert sex steroids^26^ and in the hair follicle the enzyme 5-alpha reductase type 2 (5-αR2) converts testosterone into dihydrotestosterone (DHT), which binds to intracellular AR, initiating a signaling cascade that culminates in hair thinning^14^ (Fig. 2). Compared to testosterone, DHT has approximately five times more affinity for AR.^14^

Men with 5-αR deficiency do not develop MAA, regardless of serum testosterone levels, so DHT, and consequent gene activation, is one of the main causes of the disease. The relative efficacy of 5-αR2 inhibitors in the treatment of MAA helps to corroborate this hypothesis.^27^

### Microinflammation

The limited success of therapies with androgen modulators and hair growth promoters suggests that other mechanisms may be involved in MAA.^12^

The term microinflammation is currently used to refer to the milder and more superficial inflammation that affects the follicles in MAA, in comparison with the inflammation in cicatricial alopecia.^28^

An important issue is the origin of this inflammation. The fact that it is more superficial may indicate an origin in the infundibulum and, in this case, colonization by *Propionibacterium* sp, *Staphylococcus* sp and *Malassezia* sp, among other microorganisms, could contribute with toxins and/or antigens to the initiation of the inflammatory process.^29^ Environmental factors such as irritants, pollutants, ultraviolet radiation, smoking and oxygen-free radicals can also activate the inflammatory response in follicular keratinocytes through the release of IL-1α.^30^ Adjacent fibroblasts respond to this inflammatory signal, perpetuating the cascade until the antigens are destroyed.^31^ Through DHT influence, an increase in the sebaceous gland activity could provide a more favorable microenvironment for the development of the aforementioned pro-inflammatory microorganisms.^28^

Finally, other receptors may be involved in microinflammation, such as the vitamin D receptor and retinoid receptors, as it has been shown that these have antagonistic actions on IL-8 expression and may be part of a delicate balance in the complex inflammatory cascade of hair growth.^32^, ^33^ However, it is worth noting that it remains controversial whether microinflammation in MAA is the initial trigger for its development or, even, whether it is relevant in the disease pathogenesis.^34^

## Clinical presentation and classification

**Fig. 3:**
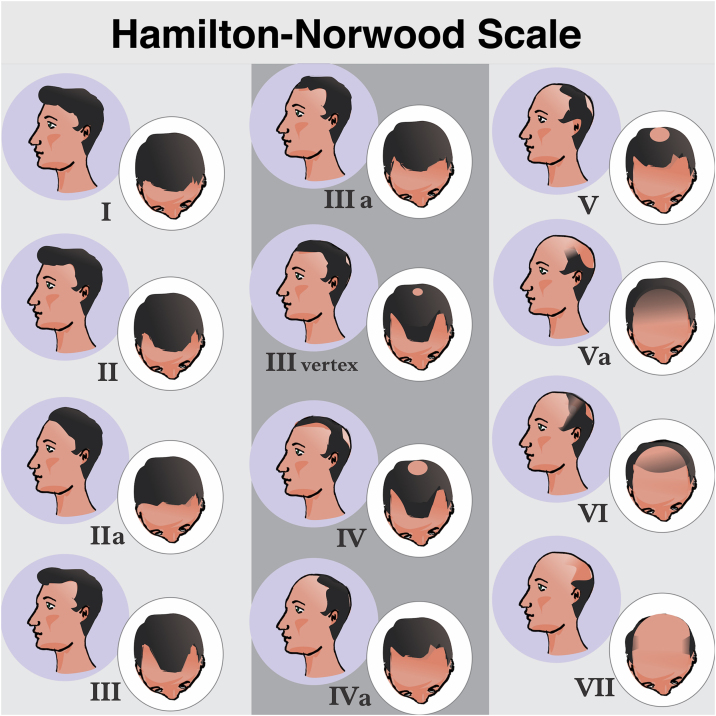
Hamilton-Norwood classification. Stage I, No apparent hair loss or minimal loss in the temporal regions; Stage II, Slight bitemporal recession, showing a symmetrical triangular shape; Stage III, Significant loss with little or no hair coverage in the temporal regions; Stage III Vertex, Hair loss is more pronounced in the vertex and the recession in the temporal region does not exceed that described in stage III; Stage IV, Significant thinning in the temporal regions and in the vertex, leaving a dense band of hair separating the two areas; Stage V, Recession of the implantation line and more evident thinning at the vertex. The band of hair separating the two regions shows even more reduced density, making the transition area less evident; Stage VI, The frontotemporal and vertex regions unite through the complete loss of the band of hair that separated them; Stage VII: More extensive form of involvement, leaving only a narrow band of hair in the lateral and occipital regions.

MAA begins with the follicular miniaturization process in the temporal regions and in the vertex. Hamilton, in 1951, classified the patterns of disease evolution.^6^ Subsequently, in 1975, Norwood modified the classification aiming to simplify it, giving rise to the most widely used of all classifications: the Hamilton-Norwood.^35^ There are seven stages that describe the evolution of the clinical picture and hair loss patterns (Fig. 3).

Forms III to V may also show subtype A, when the involvement is exclusively anterior and the band of hair separating the frontotemporal and vertex regions is not formed.

**Fig. 4:**
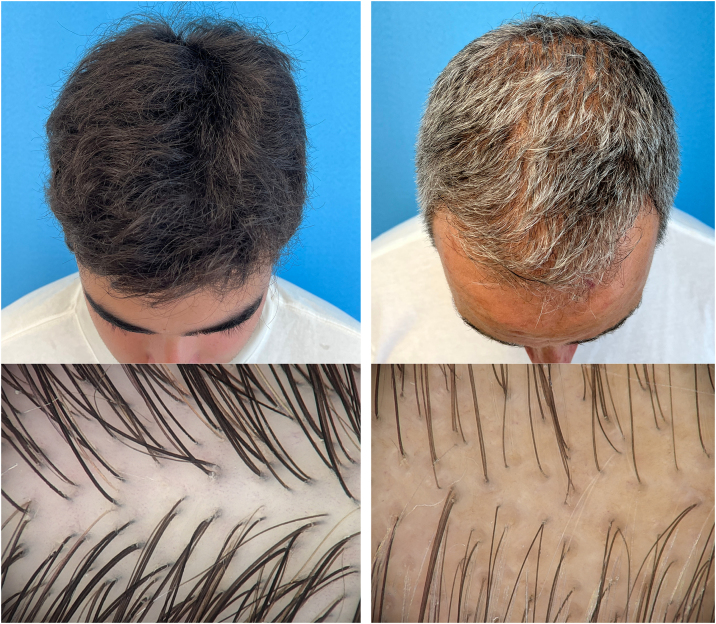
Clinical and trichoscopic comparison between father (right) and son (left): The images highlight the process of hair thinning and follicular variability characteristic of MAA, as observed in the image of the father (right).

Despite being the most widely used classification, the Hamilton-Norwood classification has limitations, such as excessive detail and inapplicability to female pattern alopecia. Lee et al. in 2007 described a new classification aiming to simplify and unify information: the Basic and Specific Classification (BASP), divided into two types: Basic, which describes the design of the implantation line, and Specific, related to severity (Fig. 4).^36^

## Diagnosis

The diagnosis of MAA is essentially clinical, with no need for laboratory tests or histopathology, which should be performed only in doubtful cases. Examination of the hair and scalp reveals the characteristic pattern of hair loss with no evidence of a scarring pattern.

In addition to the clinical picture and physical examination, the most useful test for the diagnosis of MAA is scalp dermoscopy (trichoscopy).^37^ Other less important tests in the routine will be briefly mentioned.

### Trichoscopy 38, 39

The most frequent findings in trichoscopy are based on studies in Caucasians:

• Variation in hair thickness due to progressive miniaturization, typical of MAA. This occurs because terminal hairs are gradually replaced by finer and shorter hairs (vellus hairs), except in the occipital region;
• Presence of thicker and longer hairs than typical vellus hairs, but shorter and finer than terminal hairs with a wavy appearance (wavy hair);
• The number of hairs emerging from the follicular unit decreases to one or two, characterizing a decrease in hair volume. The scalp has follicular units with two to five hair shafts and the proportion of units with only one hair is less than 30% in individuals without MAA. This information is very useful for early diagnosis;
• Yellow spots can be found in advanced cases, corresponding to keratin and sebum accumulated in the dilated ostia of the follicles;
• Presence of the peripilar sign (or perifollicular hyperpigmentation), which corresponds to the presence of a perifollicular lymphocytic infiltrate. It is observed in 22% to 60% of the patients, but it is not pathognomonic;
• Honeycomb pigmentation in Caucasian individuals with advanced MAA who have had their scalp exposed to the sun. It can be found in Caucasians with any type of non-scarring alopecia and in the healthy scalp of patients with higher phototypes.

#### Pull test

Periods of increased hair loss can be identified by a positive test during the examination.

#### Trichogram

Semi-invasive and painful test. It has been replaced by trichoscopy and is increasingly less performed in MAA.^38^

#### Phototrichogram

A non-invasive test that counts the hairs (hair density), their thickness and growth speed. It is currently more used in clinical trials.^38^

Genetic screening test

It determines whether there are genes that are susceptible to baldness.^38^

## Differential diagnoses

The differential diagnoses are described in Table 1.^40^

**Table 1:** Differential diagnoses for MAA.

Differential diagnoses
Telogen Effluvium
Alopecia Areata
Senescent Alopecia
Traction alopecia
Trichotillomania
Frontal Fibrosing Alopecia
Lichen Planopilaris
Permanent post-CT alopecia
Central Centrifugal Cicatricial Alopecia
Congenital Triangular Alopecia

## Histopathology

Histopathology is useful when the MAA pattern is not typical or for the differential diagnosis with cicatricial alopecia.

The recommended biopsy technique is a 4 mm deep punch, including the hypodermis in an area that is not completely hairless. Ideally, two biopsies should be performed in the same area of ​​alopecia, one for horizontal histological sections (in which the number of follicles, phase of the cycle and location of the inflammatory infiltrate will be assessed) and the other for vertical sections (to assess the epidermis, dermis and inflammatory process).

Two biopsies should be performed for MAA in its initial phase: one in the affected area and the second in an unaffected area to compare the size and number of follicles. In advanced cases, a single biopsy is sufficient for the diagnosis.^41^

**Fig. 5:**
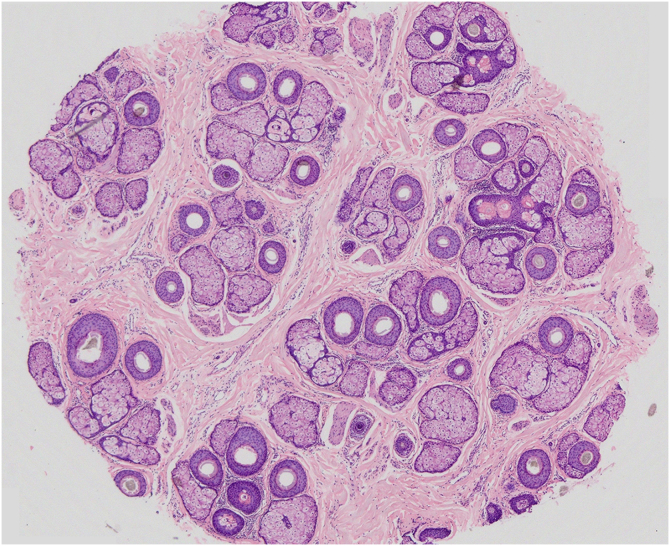
Horizontal section of a fragment of scalp skin in MAA (Hematoxylin & eosin, ×20) – 39 hair follicles at the level of the isthmus, 9 of which are vellus, and the others terminal and intermediate. Preserved follicular epithelium and sebaceous glands with enlarged lobules. All follicles are in the anagen phase, except one telogen germinal unit.

In MAA, the main histopathological characteristic is the progressive miniaturization of the terminal follicles culminating in vellus hairs. With miniaturization, the follicles ascend to the superficial dermis leading to an increase in fibrous tracts. There is an increase in the number of follicles in the telogen phase and a decrease in the anagen/telogen ratio from 7:1 up to 2:1. A mild peri-infundibular lymphocytic inflammatory process may also be observed, as well as solar elastosis in more severe cases of hair loss (Fig. 5).^42^

## Impact on quality of life

MAA has a significant impact on the quality of life of patients, especially younger men with a recent diagnosis of baldness.^43^

## Treatment

Many treatments are described for MAA, but only topical minoxidil, oral finasteride and dutasteride have been approved by the regulatory agency in Brazil (ANVISA).

### Topical and oral minoxidil

After hypertrichosis was observed in patients using oral minoxidil as an antihypertensive drug, a 2% topical formulation was developed and received approval from the US Food and Drug Administration (FDA) for the treatment of MAA in 1988. Later, in 1991, studies reported the superiority of the 5% solution when compared to the 2% solution and placebo.^44^

The mechanism of action of minoxidil in the hair follicle is not fully known and probably it’s not solely due to its vasodilatory effect. Possible effects include increased vascular endothelial growth factor in the dermal papilla, indicating that the drug induces angiogenesis; activation of prostaglandin-1-synthetase, an enzyme that promotes follicular activity; and increased expression of the hepatocyte growth factor, which promotes cell activity in the follicle.^45^

Recent data suggest that minoxidil may act on gene expression and activation of signaling pathways, leading to the upregulation of some genes. Possible antiandrogenic mechanisms have also been reported, interfering not only with AR, but also acting on two different targets related to steroid biosynthesis.^46^

In its topical presentation, it is recommended that men use the 5% solution twice a day, and for the obtained results to be maintained, treatment must be continued indefinitely.^47^ It is a safe, well-tolerated drug with few adverse effects. Among the most common are pruritus and desquamation of the scalp. Although minoxidil can cause contact dermatitis, it is propylene glycol, present in the vehicle, that most frequently causes dermatitis. In these cases, symptoms are resolved by switching to topical minoxidil without propylene glycol.

**Fig. 6:**
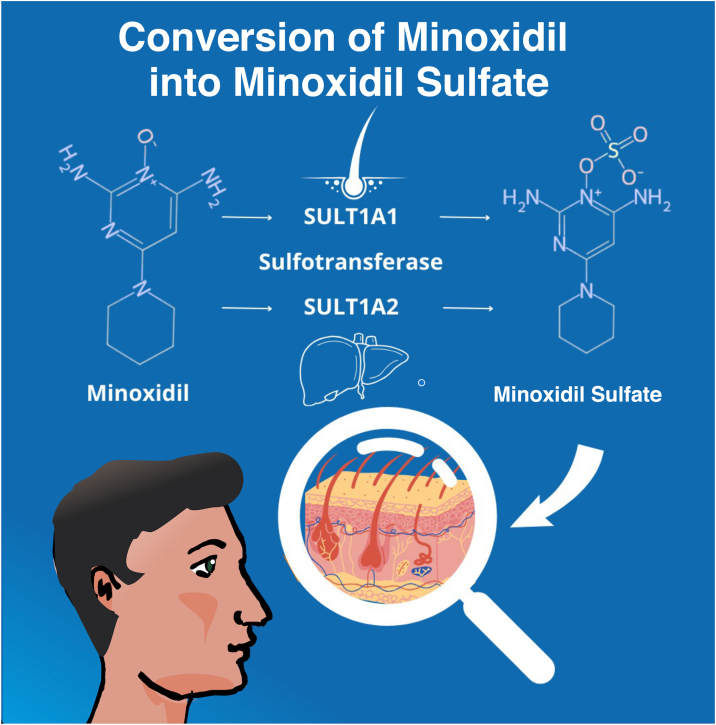
Conversion of Minoxidil into its active form, Minoxidil Sulfate.

After the topical application, the enzyme sulfotransferase (SULT1A1) in the human scalp converts minoxidil to minoxidil sulfate, which is the active form of the molecule. Differences in SULT1A1 activity between individuals may affect the efficacy of the medication, leading to inconsistencies in the response to treatment.^48^ In its oral form, minoxidil also needs to be converted to its active form, this time by hepatic sulfotransferase (SULT1A2) (Fig. 6).

Studies have demonstrated the clinical efficacy of both minoxidil 5% solution, twice daily^49^ and low-dose oral minoxidil (LDOM) with doses of 1 to 5 mg per day.^49^, ^50^ However, adherence over one year is greater with the oral medication,^51^ which may explain the perception of greater efficacy of LDOM in the dermatology office.^52^

Oral minoxidil may cause systemic side effects in a small percentage of patients, such as increased heart rate, headache, fluid retention, hypertrichosis, and edema of the lower extremities. The most commonly observed event is hypertrichosis in approximately 15% of the patients.^53^ The adverse events of oral minoxidil, however, are typically dose-dependent and reversible upon drug discontinuation. Rare and idiosyncratic, i.e., dose-independent, side effects include pericardial effusion, congestive heart failure, and allergic reactions. LDOM is likely to have a safety profile similar to the general population in patients with hypertension or arrhythmia,^54^ but cardiologic evaluation is necessary in this group.

At the beginning of use of the oral or topical forms, some patients may experience effluvium, the mechanism of which is immediate telogen release. This shedding may last from two to eight weeks and resolves spontaneously. During the first two months of treatment, an incidence of telogen effluvium of 16% was observed in the group treated with topical minoxidil, while in the group treated with oral minoxidil, this incidence was significantly lower, at only 9%.^55^

### α-reductase (5αR) inhibitors

Considered to be drugs that modify male pattern baldness, finasteride 1 mg/day and dutasteride 0.5 mg/day are approved for use.^56^ The literature shows they are effective in the treatment of patients with MAA and long-term use has shown hair growth and baldness stabilization.^57^ Their efficacy is greater in the treatment of vertex baldness than in the frontoparietal region and it is recommended that they be continued indefinitely to preserve the recovered hair.^58^ Although potentially reversible because it is a non-cicatricial alopecia, MAA in patients with significant follicle miniaturization constitutes a therapeutic challenge, and 5αR inhibitors alone have not been shown to be capable of fully reversing the disease.

Despite the well-established efficacy and mechanism of action in the treatment of MAA, including a sustained response over the years, the biggest obstacle to the oral use of 5αR inhibitors is mens insecurity about possible side effects such as decreased libido and impotence.^59^ Cohort studies report an increased risk of depression in patients treated for benign prostatic hyperplasia, especially in the first eighteen months of medication use.^60^ This is partially justified because DHT and 5αR participate in the production of several neuroactive steroids. Moreover, testosterone and DHT modulate the neuroendocrine response to stress and are inversely related to depression rates.^61^ On the other hand, the nocebo effect, that is, when a person develops symptoms and side effects from a medication simply because they have read or been warned about such a possibility, has been well documented in men using 5αR inhibitors.^62^ The choice of treatment should involve careful evaluation, avoiding the prescription in individuals with recent episodes of depression or sexual dysfunction complaints. Other adverse effects of this class of medication include orthostatic hypotension, dizziness, gynecomastia, and reduced sperm volume.^63^

Suppression of circulating DHT levels during pregnancy may inhibit the development of the external genitalia of a male fetus; therefore, pregnant women and children should avoid contact with the capsules. Men undergoing treatment with finasteride and dutasteride should not donate blood for one and six months, respectively, after the last dose to prevent administration to a pregnant transfusion recipient.^64^

Some studies have addressed the effects of 5αR inhibitors on sperm motility, morphology and volume. One study assessed the effects of daily therapy with finasteride (5 mg) and dutasteride (0.5 mg) for one year, examining serum and semen samples before and after treatment, and concluded that neither treatment had an impact on sperm morphology.^65^ There is no need to discontinue medication in men attempting to impregnate their partners or who are sexually active with pregnant partners, since the excretion of finasteride and dutasteride in semen is minimal and unlikely to cause teratogenic effects.^66^

There is a concern that 5αR inhibitors may cause or worsen underlying male infertility. There are insufficient data in the literature to categorically state their role in male fertility.

In this context, it is important to define infertility, which is a condition characterized by the inability of a couple to achieve a clinical pregnancy after 12 months of regular, unprotected sexual intercourse. Therefore, evaluating isolated male infertility outside the context of the couple attempt to become pregnant is not an approach that involves requesting a single test. In fact, male infertility is multifactorial and its incidence has been increasing over time.^67^ Requesting a spermogram may document semen characteristics below the established lower limits but does not necessarily imply infertility, and values ​​considered “normal” do not guarantee fertility.^68^

There is no standardization in the request for laboratory tests for the prescription of 5αR inhibitors. Laboratory monitoring is not necessary in patients without comorbidities.^69^ In patients over 45 years of age, after six months of treatment, it is recommended to establish a new baseline level of prostate-specific antigen (PSA).^70^, ^71^ Continuous increases in PSA during treatment may indicate prostate cancer or non-adherence to therapy. Treatment with this class of medications does not interfere with the use of PSA for the diagnosis of prostate cancer after the new baseline has been established. After stopping treatment, PSA levels return to baseline values ​​in approximately six months.

5αR inhibitors are considered by the World Anti-Doping Agency (WADA) as potential confounding factors in the assessment of athletes steroid profiles since they can interfere with the urinary excretion of several diagnostic compounds.^72^ However, given the advances in doping diagnostics, they are not included in the list of prohibited drugs by WADA.^73^

### Oral finasteride

Finasteride is a 5αR-2 inhibitor, which decreases the conversion of testosterone to DHT with a plasma half-life of 5–8 hours. After the initial peak of improvement, long-term follow-up studies demonstrate the progression of alopecia in 10.3% of cases of vertex MAA; 16.2% of the frontal type, and disease control in the biphasic or diffuse types.^58^

Recent reports of a post-finasteride syndrome, a controversial condition that can include sexual problems, depression and anxiety, have created fear among patients seeking treatment. While studies neither confirm nor refute the syndrome as a valid disease, the safe long-term use of oral finasteride over a period of ten years has been documented.^74^

### Oral dutasteride

Dutasteride is an inhibitor of 5αR-1 and 2, with a half-life of five weeks, which is three times more potent in inhibiting 5αR-2 than finasteride and 100 times more potent in inhibiting 5α-1.^75^ These characteristics justify the superior effect of dutasteride 0.5 mg/day when compared to finasteride 1 mg/day in the treatment of MAA,^76^ without showing, to date, significant differences in adverse effects (Table 2).

**Table 2:** Comparison between Finasteride and Dutasteride.

Differences between 5α-reductase inhibitors		
	Finasteride	Dutasteride
Half-life	5-8 hours	3-5 weeks
5-AR Inhibition	Only type 2	Types 1 and 2
Systemic DHT suppression	∼71%	>90%

### Topical finasteride

In an attempt to mitigate its systemic effects, finasteride in topical preparations has been studied and has shown the ability to reduce DHT levels in both plasma and scalp.^77^ The lack of standardized concentrations and vehicles makes it unpredictable to determine the efficacy of this route of administration.

### Topical dutasteride

Due to the large molecular size of dutasteride, it is difficult to formulate and administer it as a topical agent. On the other hand, its large size and lipophilic nature may contribute to its persistence on the scalp, with reduced systemic absorption. At present, however, there is insufficient data in the literature to support its usefulness in the treatment of MAA.^78^

### Adjuvant treatments

#### Scalp intradermotherapy

Intradermotherapy (often referred to as mesotherapy) is performed by injecting a medication or a mixture of several substances into the scalp. Although it has been widely used, the choice of doses and mixtures is based on personal experiences. Few treatments are supported by evidence from scientific studies.

Among the medications used through this route of administration, dutasteride stands out due to its increasing use and scientific publications. This circumvents the epidermal barrier and minimizes possible adverse events.^79^ There is no standardization regarding the frequency of application. The main adverse event is pain, which can be overcome with anesthetic blocks. Less commonly, edema of the forehead or even the face and hematomas may occur. According to current knowledge, more studies are still needed to evaluate its therapeutic efficacy when compared to the already established oral route.^80^

There is limited evidence on intradermal therapy with finasteride and minoxidil. The use of vitamins such as biotin has not been shown to be effective.^70^

Rare adverse events have been described, including cases of alopecia secondary to injectables, which present clinically mimicking alopecia areata and/or cicatricial alopecia.^81^ Different substances can cause hair loss due to inflammation, damaging hair follicles through mechanisms that are not yet fully understood.

Microinfusion of drugs into the skin (MMP®) combines the delivery of drugs with the potential benefit of a smaller dose and more homogeneous distribution.^82^ Although this technique is widely used in Brazil, only case reports have been published.

#### Microneedling

Microneedling is a minimally invasive procedure in which percutaneous injury is induced with microneedles, resulting in the release of several growth factors to induce collagen remodeling. These factors potentially promote angiogenesis, wound healing, and reverse fibrosis.^83^ A review of 17 studies evaluated the improvement of androgenetic alopecia (AGA) and alopecia areata with microneedling using solutions such as 5% minoxidil and/or platelet rich plasma (PRP). Most clinical trials showed positive results; however, the level of evidence in the studies and the heterogeneity of the methods do not allow the evaluation of long-term efficacy.^84^ Microneedling is often used as an adjuvant treatment and should not be indicated as monotherapy, since it has been shown that it does not prevent disease progression in MAA.^85^

Pain, hematoma, and folliculitis may be observed with the technique, similar to other procedures involving injections (intradermal therapy).

#### Photobiomodulation: low-level laser therapy and LED

Photobiomodulation (PBM) therapy uses low-irradiance lasers or light emitting diodes (LEDs) to promote cell biostimulation without producing thermal effects. The mechanism of action is not fully understood; however, it is believed that absorption of red light by cytochrome c oxidase in mitochondria leads to photodissociation of inhibitory nitric oxide, which results in increased ATP production, modulation of reactive oxygen species, and induction of transcription factors.^86^

In 1967, Endre Mester tested lasers on mice, looking for the potential to develop cancer. Surprisingly, mice treated with low-level lasers did not develop cancer and showed accelerated hair growth.^87^ Moreover, paradoxical hypertrichosis, i.e., hair growth in areas treated with low-dose lasers for hair removal, has been reported, supporting the photobiomodulation mechanism.^88^

For the treatment of MAA, the most commonly used wavelengths are around 655 nm.^89^ A meta-analysis compared different devices and found no differences in efficacy between the presentation forms (comb, helmet or cap) but favored laser devices over LED.^90^

Although the mechanism of action of low-intensity laser therapy (LLT) in MAA has a theoretical basis, the clinical impact as an isolated therapy on hair coverage is small. The new hairs detected in phototrichograms are probably miniaturized and short, resulting in limited benefit when used as monotherapy. However, laser use represents a safe option as adjuvant therapy.^90^

#### Fractional lasers and other technologies

Ablative and non-ablative fractional lasers and microneedling with radiofrequency have also been used, especially to increase drug penetration (Laser and Energy Assisted Drug Delivery ‒ LEADD).^91^ When compared to the use of laser as monotherapy, LEADD did not show superiority. Considering that both treatments improved the assessed alopecia criteria, one can assume that there are mechanisms specific to laser in hair growth modulation; however, further studies are needed to corroborate these findings.^91^

#### Platelet-rich plasma

Intradermotherapy with autologous platelet-rich plasma (PRP) is considered an adjuvant in MAA therapeutic regimens. The exact mechanism by which it induces hair growth is unknown. Platelet-derived growth factors released during the procedure can lead to increased expression of type I collagen and matrix metalloproteinase 1 mRNA in dermal fibroblasts, increased upregulation of Bcl-2, an anti-apoptotic protein, and activation of β-catenin and FGF-7 in dermal fibroblasts, helping to prolong the anagen hair phase.^92^ This set of combined effects can contribute to increased density and follicular regeneration. An additional effect of PRP has been demonstrated over the reference standard therapy, Minoxidil 5% and finasteride 1 mg daily.^93^

In Brazil, it is considered an experimental therapy linked to research protocols approved by Research Ethics Committees.^94^ Despite the favorable outcomes in increasing hair thickness and density when using PRP in MAA,^95^ a recent meta-analysis showed there is insufficient scientific evidence to conclude its effectiveness due to the great heterogeneity of parameters used to evaluate the technique.^96^

### Less established therapeutic approaches

#### Nutraceuticals

Micronutrients play an essential role in the follicular cycle, due to the high metabolism and accelerated cell renewal that occurs in the follicles.^97^, ^98^

Oral regimens are easy for patients to accept, but many still lack proof of efficacy and their prescription should be individualized, considering the risk-benefit of supplementation. Multivitamins are sold without a prescription and there is a concern about the delay in the correct diagnosis of scalp diseases caused by the indiscriminate use of these medications. To date, there is insufficient data in the literature to support the supplementation of any substances, except in cases of laboratory-proven deficiency.

While biotin (vitamin B7) deficiency can lead to alopecia, rash, and brittle nails, the supplementation of this vitamin in patients without deficiency has not been shown to be effective for hair growth.^99^ Patients considered at risk for biotin deficiency include those who ingest high quantities of raw eggs, intestinal malabsorption syndromes, alcoholism, pregnancy, prolonged use of antibiotics, and medications such as valproic acid and isotretinoin.^100^ Considering this deficiency is extremely rare in the general population, supplementation should not be routinely recommended, as it can lead to errors in several laboratory tests.^101^

Vitamin D modulates the growth and differentiation of keratinocytes through its binding to a nuclear receptor. In cases of mutation in this receptor, there is resistance to vitamin D, characterized among other signs, by hair thinning and alopecia. Vitamin D supplementation in patients without this mutation has already been studied in non-cicatricial alopecia and has shown conflicting results.^98^

Although they are important factors in protein synthesis and cell growth, respectively, excess selenium and vitamin A are associated with hair loss and should also not be routinely replaced.^102^ Hypervitaminosis A is a risk in these situations and can lead to toxicity.^95^

Iron deficiency is common, especially in women, but is not necessarily associated with an increased risk of androgenetic alopecia or telogen effluvium.^103^

Zinc deficiency can also lead to alopecia, but a stronger correlation has been seen with alopecia areata and telogen effluvium compared to androgenetic alopecia, and more studies are required to assess their efficacy in the treatment of the latter condition.^103^

#### Botanical extracts

Saw palmetto (*Serenoa repens*), green tea (*Camellia sinensis*), pumpkin seed (*Cucurbita pepo*), and licorice (Glycyrrhiza glabra) are herbal remedies that may have an inhibitory action on the 5αR enzyme and, therefore, could eventually be used in the treatment of MAA. Larger and comparative studies with 5αR inhibitors established as reference are needed to better characterize their degree of efficacy and safety profile.^104^

#### Ketoconazole

In addition to its antifungal and anti-inflammatory properties against *Malassezia* for the treatment of seborrheic dermatitis, antiandrogenic properties have been described for ketoconazole, with a possible DHT inhibition mechanism.^105^ Lotions or shampoos at 2% can be applied to the scalp as adjuvant therapy. More robust studies are needed to better characterize the mechanism of action and efficacy.^106^

#### Latanoprost/Bimatoprost – prostaglandin analogs

The use of prostaglandin analogues for alopecia was suggested when hair growth of the eyebrows and eyelashes was observed in patients with glaucoma using eye drops based on these drugs. Prostaglandin F2 (PGF2) and prostaglandin E2 (PGE2) induce hair growth and prolong the anagen phase.^107^ Two published studies with latanoprost suggest that this medication is effective at doses ranging from 0.005% to 0.1%; however, the results are still inferior to those of topical minoxidil. For this reason and because these medications are expensive, they are currently scarcely used for the treatment of MAA.^108^, ^109^

#### Restoration surgery

In MAA, hairless areas can be permanently covered again cosmetically, although with reduced density. Hair restoration surgery involves scalp reduction surgery, hair transplantation, or a combination of both.

In recent decades, hair transplantation has evolved into a microsurgical procedure in which follicular units of 1 to 4 hairs are transplanted in large numbers and at high density. There are currently two techniques for extracting follicular units: follicular unit extraction (FUE) and follicular unit transplantation (FUT). In the FUT technique, a strip of scalp is removed from the occipital region, the follicular units are separated, lapidated and implanted in the recipient region. This procedure leaves a linear scar in the donor region. In the FUE technique, the follicular units are extracted through micropunch, manually or with automated devices, and subsequently implanted, similar to what occurs with FUT. The difference is that, in this procedure, the resulting scars are punctiform and scattered throughout the donor area and may be practically imperceptible.^110^

Hair restoration surgery is not a cure for baldness; therefore, even after the procedure, it is necessary to continue treatment for the disease, otherwise, the condition may progress and result in loss of the cosmetic result, due to the progressive miniaturization of the native hairs in the new region. Moreover, it is important to exclude conditions in which the transplant may be contraindicated, such as body dysmorphism, unrealistic expectations, alopecia areata and others in which the procedure will not bring aesthetic benefits, such as in the case of insufficient donor areas and non-controlled inflammatory cicatricial alopecia.^111^

## Conclusion

MAA is a long-known disease, with its clinical characteristics, diagnostic parameters, therapeutic options and efficacy analysis instruments in constant evolution, and the evidence presented herein depicts the most recent advances.

In this review, some non-standardized therapeutic approaches, with efficacy and safety analysis of insufficient level of evidence, were not mentioned.

MMA treatment should be personalized since one of the most important factors for therapeutic success is improving adherence through an individualized therapeutic strategy. When using off-label drugs or therapies, the professional must inform the patient and obtain the consent (verbal or written), and this must be recorded in the medical file. The evaluation of the efficacy of a given treatment must be performed after at least six to 12 months of use.

## Financial support

None declared.

## Authors’ contributions

Gabriel Lazzeri Cortez: Design and planning of the study; collection of data, or analysis and interpretation of data; drafting and editing of the manuscript or critical review of important intellectual content; effective participation in research orientation; critical review of the literature; approval of the final version of the manuscript.

Karime Hassun: Design and planning of the study; a collection of data, or analysis and interpretation of data; drafting and editing of the manuscript or critical review of important intellectual content; effective participation in research orientation; critical review of the literature; approval of the final version of the manuscript.

Luciana Ribeiro Patricio Linhares: Design and planning of the study; collection of data, or analysis and interpretation of data; drafting and editing of the manuscript or critical review of important intellectual content; effective participation in research orientation; critical review of the literature; approval of the final version of the manuscript.

Verena Florenço: Design and planning of the study; collection of data, or analysis and interpretation of data; drafting and editing of the manuscript or critical review of important intellectual content; effective participation in research orientation; critical review of the literature; approval of the final version of the manuscript.

Maria Valeria Bussamara Pinheiro: Design and planning of the study; collection of data, or analysis and interpretation of data; drafting and editing of the manuscript or critical review of important intellectual content; effective participation in research orientation; critical review of the literature; approval of the final version of the manuscript.

Mauricio Mendonça do Nascimento: Design and planning of the study; collection of data, or analysis and interpretation of data; drafting and editing of the manuscript or critical review of important intellectual content; effective participation in research orientation; critical review of the literature; approval of the final version of the manuscript.

## Conflicts of interest

None declared.
